# Expression and Distribution of Calcium-Binding Protein S100P in Human Placenta during Pregnancy

**DOI:** 10.22074/ijfs.2015.4189

**Published:** 2015-02-07

**Authors:** Hai-Yan Zhu, Xiao-Mei Tong, Xiao-Na Lin, Ling-Ying Jiang, Jun-Xia Wang, Song-Ying Zhang

**Affiliations:** Assisted Reproduction Unit, Department of Obstetrics and Gynecology , Sir Run Run Show Hospital, College of Medicine, Zhejiang University, Hangzhou, China

**Keywords:** S100P, Placenta, Trophoblast, Pregnancy

## Abstract

**Background:**

S100P is a member of the S100 family of calcium-binding proteins, and it participates in pathophysiological events, such as tumor growth and invasion. Based on the striking similarities between trophoblast cells and tumor cells with regard to proliferative and invasive properties, we raised the question of whether and how S100P expresses in trophoblast cells during development. This study aimed to investigate the expression pattern of S100P in the human placenta during pregnancy development.

**Materials and Methods:**

In this experimental study, we collected 16 first-trimester placental tissues, 10 second-trimester placental tissues, and 12 term placentas. The mRNA expression levels of S100P were detected by reverse-transcription-polymerase chain reaction (RT-PCR) and quantitative real-time PCR, the protein expression levels were detected by western blot, and the localization of S100P was measured by immunohistochemical staining. The values obtained from PCR and western blot analysis were expressed as the mean ± SD. Levene’s test was used to test equal variances, and one-way analysis of variance (ANOVA) was used to evaluate differences between groups.

**Results:**

Protein and mRNA expression of S100P could be detected in placenta during pregnancy, with minor higher levels in first-trimester (p>0.05). Immunohistochemical staining revealed that S100P protein was strongly expressed in syncytiotrophoblasts, and moderate expression was detected in villous cytotrophoblasts and cytotrophoblast columns. The S100P protein was localized to both cytoplasm and nuclei in syncytiotrophoblasts, while it only existed in the cytoplasm of cytotrophoblasts.

**Conclusion:**

S100P was strongly detected in human placenta during pregnancy. The specific expression and distribution of S100P in human placenta throughout gestation suggested that S100P function might vary with its location in the placenta.

## Introduction

S100 molecules are small calcium-binding proteins that display 30-50% protein structure homology within their family. The S100 subfamily shares a common Ca^2+^-binding structural motif: the EF-hand (1, 2). More than 21 members of this subfamily have been identified to date, with functions related to cell proliferation, differentiation, adhesion, and apoptosis (3, 4). S100P is a relatively small (95-amino acid) isoform of the S100 protein family that was first purified from the placenta (5, 6), which suggests that it may be related to pregnancy, and a number of studies have revealed that S100P plays a role in many cancers, such as breast, pancreatic, and lung carcinomas (7-10).

The placenta is a remarkable organ, which provides critical transport functions between the maternal and fetal circulations during fetus development. Normal development and function of the placenta is critical to achieving a successful pregnancy, as normal fetal growth depends directly on the transfer of nutrients from mother to fetus via this organ (11).

Placenta is the original tissue in which S100P was described for the first time. However, there are no available data concerning the expression and localization of S100P during the course of pregnancy, and in particular during first-trimester gestation. To address these points, we analyzed the expression and distribution of S100P in human placenta. The aim of the present study was to characterize S100P in human placenta during first-trimester gestation, second-trimester gestation, and at term using reverse transcription-polymerase chain reaction (RT-PCR), real-time PCR, western blot, and immunohistochemical techniques.

## Materials and Methods

### Tissue collection

This study was an experimental study. Patients attending the Assisted Reproduction Unit, Department of Obstetrics and Gynecology, Sir Run Run Shaw Hospital, College of Medicine, Zhejiang University, Hangzhou, China, for treatment were invited to participate in the study. The clinical characteristics of patients are stated in table 1.

The inclusion criteria were 22-35 years of age, regular menstrual periods with a cycle length between 26 and 35 days, and physical health. The exclusion criterion was a history of any kind of pharmacotherapy, hypertensive disorders, and fetal growth restriction. Samples of placental tissue of the first trimester (n=16) were obtained from healthy women undergoing suction termination of pregnancy (6-9 weeks) for inevitable abortion. Placental tissue of the second trimester (n=10) was taken from healthy women undergoing induced labor as a result of inevitable abortion or fetus cheilognathopalatoschisis. Term placentas (n=12) were collected after an uncomplicated pregnancy and vaginal delivery. All of the samples were rinsed in sterile phosphate-buffered saline (PBS) to remove blood and debris, and collected in -80˚C or formalin.

**Table 1:** Clinical characteristics of the patients and expression level of S100P mRNA


Group	Age (Y)(Mean ± SEM)	Menstrual cycle	Reason for termination	mRNA of S100P/GAPDH(Mean ± SEM)

**First-trimester placenta**	26.6 ± 3.0	Regular	Inevitable abortion	33.582± 7.001*
**Second-trimester placenta**	26.8 ± 3.2	Regular	Cervical incompetence or fetuscheilognathopalatoschisis	25.488± 5.854
**Full-term placenta**	27.3 ± 3.6	Regular	No medical reasons	30.014 ± 6.922


The three groups contain 16, 10, and 12 samples, respectively. Data are presented as mean ± SEM of normalized expression values against internal controls (GAPDH mRNA). *; P>0.05. We analyzed the data using Levene’s test and one-way ANOVA. The level of significance was set at p<0.05.

### RNA extraction and RT-PCR

Total RNA was extracted using TRIzol (Invitrogen, Carlsbad, CA, USA). Total RNA (2 μg) was used for first-strand complementary DNA (cDNA) synthesis in a 25-μl reaction volume. The cDNA was then amplified by RT-PCR. The reactions were prepared as follows: 5 μl of 5× reaction buffer, 1 μl oligo (dT), 1 μl deoxy-NTP mix (10 mmol/l each), and 1 μl Moloney murine leukemia virus reverse transcriptase. The PCR reactions were prepared containing: 2 μl of cDNA, 2 μl of 25 mmol/l MgCl_2_, 1 μl of 10 mmol/l dNTPs, 0.2 μl of Taq DNA polymerase (5 U/μl), 1 μl of 0.25 mmol/l specific primers, 2.5 μl of 10× reaction buffer and double-distilled water to a final volume of 25 μl.

A 5-minute pre-cycle at 95˚C was followed by 30 cycles of 30 seconds at 95˚C, 30 seconds at 56˚C, and 30 seconds at 72˚C. After the final cycle, the samples were kept at 72˚C for 4 minutes to complete the synthesis. The PCR products were then separated by electrophoresis in a 1.0% agarose gel stained with ethidium bromide and visualized using an imaging analysis system. The intensities of bands of S100P were normalized to glyceraldehyde-3-phosphate dehydrogenase (GAPDH), which was amplified simultaneously. The primer pairs for cDNA amplification were as follows: S100P, 5΄-ATGACGGAACTAGAGACAGCCATGGGC-3΄ and 5΄- GGAATCTGTGACATCTCCAGGGCATCA-3΄; and GAPDH, 5΄-TGACTTCAACAGCGACACCCA-3΄ and 5΄-CACCCTGTTGCTGTAGCCAAA-3΄.

### Real-time PCR

Thirty-eight tissues were selected for real-time PCR analysis, with GAPDH as a control. Total RNA, 2 μg, was used to generate cDNA. The PCRs contained the following: 4 μl of cDNA, 4 μl of 10× reaction buffer, 0.8 μl of 10 mmol/L dNTP, 0.3 μl of 5 U/mL Taq DNA polymerase, 3.2 μl of 25 mmol/L MgCl_2_, 0.3 μl of probe, 1 μl of specific primers, and 26.4 μl of double-distilled water. The PCR was performed by monitoring in real time the increase of fluorescence of SYBR green I dye (Takara, Shiga, Japan) with a Rotor-Gene 3000 (Corbett Research, Sydney, Australia). The relative copy number of each transcript was calculated by the concentration-CT standard curve method and normalized using the average expression of GAPDH.

#### Western blot analysis

Total protein was separated using sodium dodecyl sulfate-polyacrylamide gel electrophoresis (SDS-PAGE). The separation gel concentration was 15% and the voltage was 80-100 V. Then the gel was transferred to polyvinylidene fluoride membranes using an electroblotting apparatus for 1 hour. Membranes were blocked in 5% non-fat dried milk in Tris-buffered saline (TBS) with Tween 20 (TBS-T) for 1 hour at room temperature. The polyvinylidene fluoride (PVDF) membranes were incubated with rabbit anti-S100P at 1:1000 (P25815, Epitomics, Burlingame, CA, USA) for 2 hours at room temperature. Membranes were then incubated with horseradish peroxidase and goat anti-rabbit IgG at 1:5000 for 1 hour at room temperature. Chemiluminescence was detected using an ECL Chemiluminescent Substrate Kit, according to the manufacturer’s instructions (Millipore, Boston, MA, USA). GAPDH was used as an internal control. The protein level of S100P was normalized to that of GAPDH.

#### Immunohistochemistry

Placental tissues were fixed in formalin, embedded in paraffin, and sectioned (4 μm thickness). The tissue sections were de-paraffinized in xylene, dehydrated in graded alcohols, and placed in buffered saline. Subsequently, antigen retrieval was achieved by heating the slides in a microwave oven in 10 mmol citrate buffer. After antigen retrieval, sections were incubated with an aqueous solution of 3% hydrogen peroxide followed by incubation with 5% non-fat milk, which serves as a blocking agent for nonspecific binding, for 20 minutes at 37˚C.

After rinsing the slides in buffer for 5 minutes, slides were incubated with the rabbit anti-S100P (P25815, Epitomics, USA; diluted 1:500 in PBS), anti-cytokeratin 7 antibody (Zhongshan, Beijing, China; diluted 1:200 in PBS), anti-vimentin (Zhongshan, Beijing, China; diluted 1:200 in PBS) for 30 minutes at 37˚C. The anti-cytokeratin-7 antibody was positive for trophoblast cells, while the anti-vimentin antibody was positive for stromal cells. For S100P, using secondary antibody alone was applied as negative control. After washing, the secondary biotinylated antibody was then applied for 30 minutes at room temperature. After rinsing with PBS, an avidin-biotin-peroxidase complex (Vectastain Elite ABC kit, Vector Laboratories Inc., Burlingame, CA, USA) was added to the sections and incubated for 1 hour at room temperature. Then, 3, 3΄-diaminobenzidine (DAB) peroxidase substrate kits (Vector Laboratories Inc., Burlingame, CA, USA) were used to visualize the immunostaining and hematoxylin was used to counterstain.

### Statistical analysis

Each experiment was repeated at least three times. The values obtained from PCR and western blot analysis were expressed as the mean ± SD. Statistical analysis was carried out using the Statistical Package for the Social Sciences (SPSS; SPSS Inc., Chicago, IL, USA) version 11.0. Levene’s test was used to test equal variances, and one-way analysis of variance (ANOVA) was used to evaluate differences between groups. Differences were considered significant at p<0.05.

### Ethical considerations

This study was approved by the Ethics Committee of the College of Medicine, Zhejiang University, China. Informed consent was obtained from all patients.

## Results

### The mRNA expression of S100P in placenta during pregnancy

**Fig 1:**
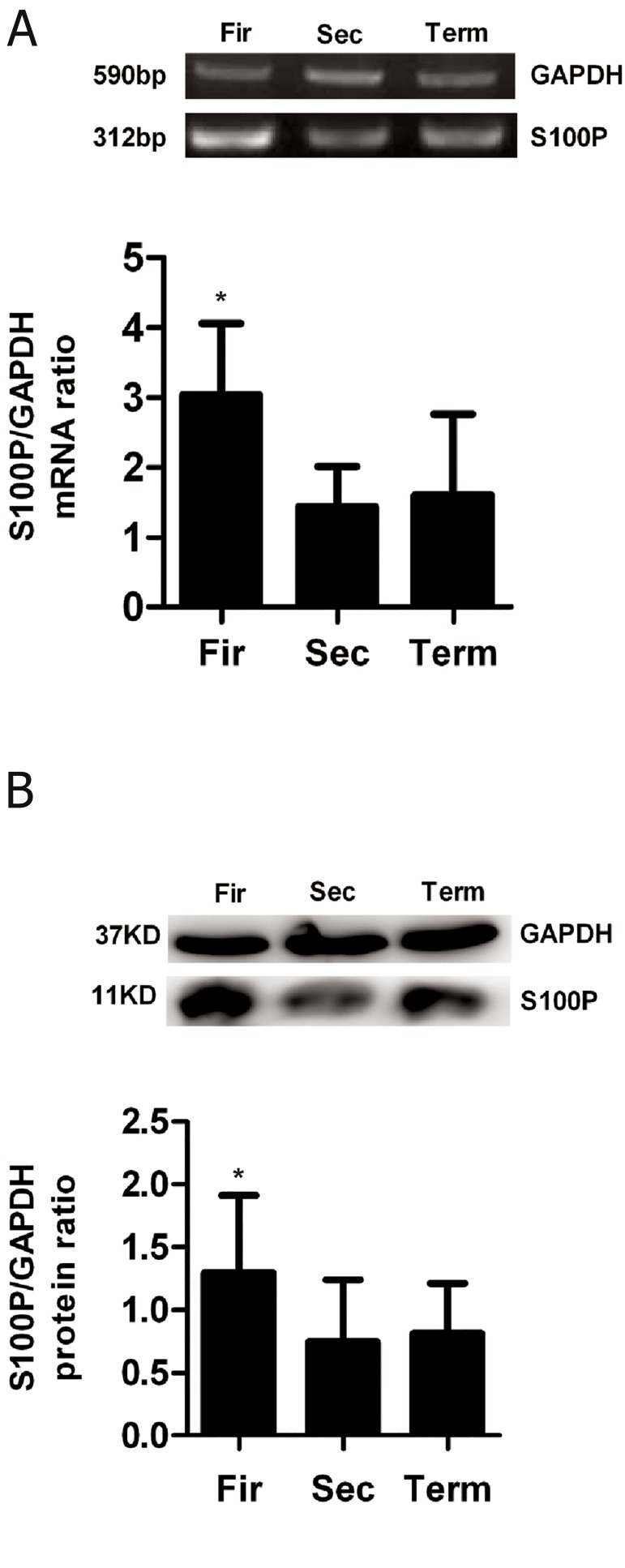
Expression of S100P mRNA in placenta during pregnancy obtained by RT-PCR (A), and protein expression of S100P was detected by western blot (B). Fir; First-trimester placenta, Sec; Second-trimester placenta, Term; Full-term placenta and *; P>0.05. Each bar is the mean ± SEM, n=3 separate experiments.

In the present study, we applied RT-PCR and real-time PCR to detect the expression of S100P mRNA in human placental tissue. S100P mRNA expression was detected in placental tissues from the first-trimester gestation, second trimester, and at term (Fig 1A), and the expression levels were a little higher in the first trimester than those in the second trimester and at term (p>0.05, Table 1).

### The protein expression of S100P in placenta during pregnancy

S100P protein expression was analyzed by western blot on solubilized protein extracts from placental tissue. A band of 11 kDa was found in placental tissue (Fig 1B). Compared with second trimester and term, the band signal increased a little in the first trimester (p>0.05).

### Immunohistochemistry for S100P in first-trimester, second-trimester, and term placental tissue

**Fig 2:**
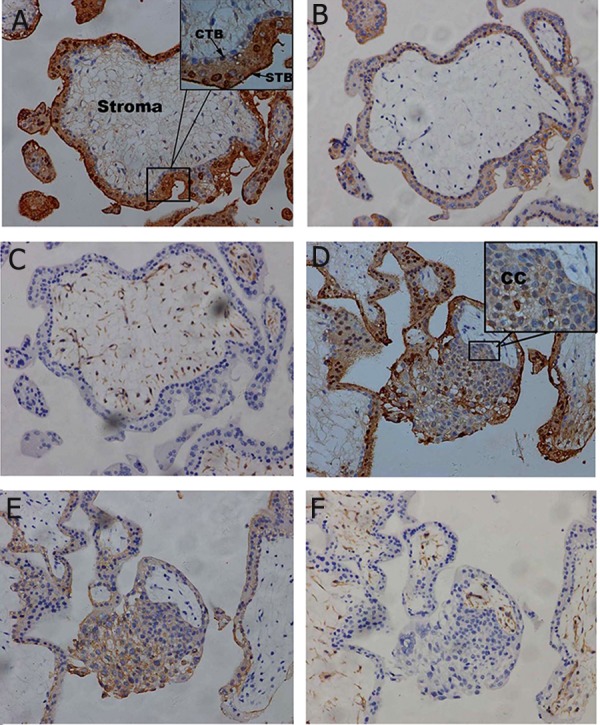
In first-trimester placenta, S100P immunopositive staining showed an intense signal in the cytoplasm and nuclei of syncytiotrophoblasts, whereas villous cytotrophoblasts (A) and cytotrophoblast columns (D) were stained at the cytoplasm. Furthermore, stromal cells were also stained by S100P antibodies (A). Trophoblasts were immunoreactive to cytokeratin-7 (B, E) and villous stroma was positive to vimentin (C, F). Figures show as ×200 magnification. CTB; Cytotrophoblasts, STB; Syncytiotrophoblasts and CC; Cytotrophoblast columns.

**Fig 3:**
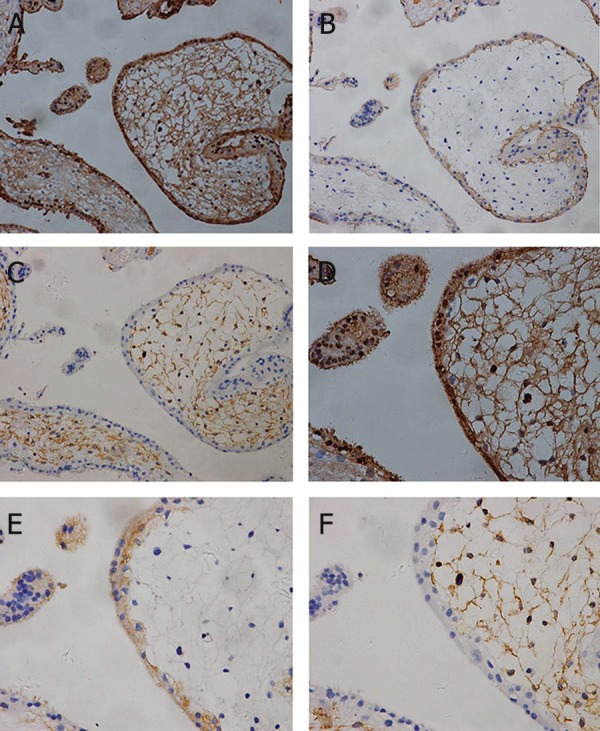
In second-trimester placenta, an intense signal was still shown in syncytiotrophoblasts and stromal cells (A, D). Anti-cytokeratin-7 antibody was positive for trophoblast cells (B, E), and anti-vimentin antibody was positive for villous stroma (C, F). A, B, C: ×200 magnification and D, E, F: ×400 magnification.

**Fig 4:**
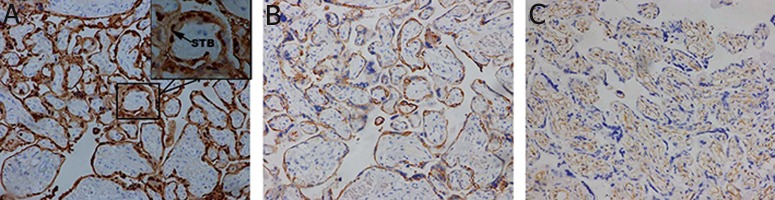
In placenta at term, S100P immunoreactivity was still localized at the cytoplasm and nuclei of syncytiotrophoblasts, whereas the villous stroma was stained weakly or negative for S100P (A). Trophoblasts were immunoreactive to cytokeratin-7 (B) and villous stroma was positive to vimentin (C). STB; Syncytiotrophoblasts. Figures show as ×200 magnification.

In first-trimester placenta, S100P immunopositive staining showed an intense signal in the cytoplasm and nuclei of syncytiotrophoblasts, whereas villous cytotrophoblasts (Fig 2A) and cytotrophoblast columns (Fig 2D) were stained at the cytoplasm. Furthermore, stromal cells were also stained by S100P antibodies (Fig 2A). In second-trimester placenta, an intense signal was still shown in syncytiotrophoblasts and stromal cells (Fig 3A). In placenta at term, S100P immunoreactivity was still localized at the cytoplasm and nuclei of syncytiotrophoblasts, whereas the villous stroma was stained weakly or negative for S100P (Fig 4A). From the first trimester to term, trophoblasts were immunoreactive to cytokeratin-7 (Figes2B, E, 3B, 4B) and villous stroma was positive to vimentin (Figes2C, F, 3C, 4C).

## Discussion

S100P can be detected in placenta (5, 12). However, there are, to date, no reports describing the expression and distribution of S100P in human placenta during the entire pregnancy-and certainly not the cell type involved. The present study showed, for the first time, an expressional profile of S100P protein in human placental tissue during the course of pregnancy. Our data demonstrated that the increased expression levels of S100P were detected throughout gestation, with minor higher levels in first-trimester placenta. S100P protein was strongly expressed in the syncytiotrophoblasts, and S100P immunoreactivity was abundantly present in syncytiotrophoblast cytoplasm and nuclei. Moreover, moderate expression was also found in villous cytotrophoblasts and cytotrophoblast columns.

S100P is one of the least studied members of the S100 family. It is an 11-kDa protein and was originally isolated from the placenta. S100P expression was then detected in tumor cell lines and carcinomas derived from the prostate, breast, colon, pancreas, and other tumor types, and it is associated with immortalized, malignant, hormone-independent, and chemoresistant phenotypes (13-15). Convincing evidence has shown that S100P expression is found in a variety of cancers and appears to be correlated with the invasion and metastatic process of tumor cells (13, 16-18). We have previously reported that S100P is highly expressed during the implantation window in human endometrium (19), which suggests that S100P is associated with endometrial receptivity.

In the present study, in first-trimester placenta, S100P was moderately expressed in the cytoplasm of villous cytotrophoblasts and cytotrophoblast columns, and was found in the cytoplasm and nuclei of syncytiotrophoblasts. Furthermore, in term placenta, the striking localization of syncytiotrophoblasts was still invariant.

The villous cytotrophoblasts are proliferative and capable of fusing to form the multinucleated syncytiotrophoblast. The column cytotrophoblasts actively proliferate and tightly anchor the placenta to the uterine wall; in addition, they can further differentiate into invasive extravillous trophoblasts to remodel the uterine decidua and spiral arteries (20). Trophoblast proliferation and invasion constitute a series of tightly controlled processes that are pivotal to implantation and placentation. The proliferation of trophoblast cells is prominent in the first trimester of pregnancy. The present data, showing absence of nucleus staining and moderate cytoplasm staining of cytotrophoblasts and cytotrophoblast columns, suggest that cytotrophoblast can produce S100P, but S100P does not participate in its nucleus transcription. As the early pregnant villous cytotrophoblasts and column cytotrophoblasts are highly proliferative, their higher expression in first-trimester placenta indicates that S100P may be important for cytotrophoblast proliferation. Further research is needed to explain whether S100P has any role in cytotrophoblast proliferation and invasion.

As the placenta developed, the cytotrophoblasts and cytotrophoblast columns gradually disappeared. Syncytiotrophoblasts became the main kind of trophoblast cells at term. Syncytiotrophoblasts were differentiated from cytotrophoblasts. The present findings, showing strong cytoplasm and nuclei-staining syncytiotrophoblasts, suggest that, upon differentiation, syncytiotrophoblasts acquire S100P synthesis and transport capabilities. The component of S100P in the nucleus might bind to S100P binding protein (S100PBP), the exact role of which remains unclear. The syncytiotrophoblast in the placenta serves as an interface for efficient maternal-fetal exchange of nutrients, metabolites, and xenobiotic compounds, and membrane transport processes play pivotal roles in the efficient uptake of certain compounds and in the exclusion of others (21). Syncytiotrophoblasts are responsible for hormone production (20). The change of S100P expression as cytotrophoblast differentiation may tell us S100P have an important role in syncytiotrophoblasts. Whether the presence of S100P relates to the syncytiotrophoblast function or not remains elusive.

## Conclusion

The present data show an intimate pattern of distribution of S100P in placenta, and demonstrate that the expression of S100P is strongly detected in human placenta during pregnancy. Further investigation is required to study the roles and associated molecular mechanisms of S100P in normal placenta.
